# AGING AND THE LIVED EXPERIENCES OF TRANSGENDER AND GENDER NONCONFORMING OLDER ADULTS

**DOI:** 10.1093/geroni/igad104.2569

**Published:** 2023-12-21

**Authors:** Jeanne Koller, Paul Urbanski

**Affiliations:** Monmouth University, West Long Branch, New Jersey, United States; Monmouth University, West Long Branch, New Jersey, United States

## Abstract

Adults identifying as transgender and gender non-conforming (T/GNC) in the United States may experience adversity, victimization, and discrimination across the life span (McDowell, et al., 2019). T/GNC older adults have higher rates of poverty, homelessness, social isolation, depression, and violence compared to older adults in the general population and compared with lesbian, gay, and bisexual (LGB) older adults (Sloan & Benson, 2022). This descriptive phenomenological study utilized a purposive and snowball sampling strategy and sought to increase understanding of this diverse group. Twenty participants aged 50 and older were interviewed. Themes included fear and/or actual loss of relationships after “coming out” as transgender; concerns and complexity of healthcare and anticipated changes in healthcare if faced with needing long-term care; navigating gendered space; living as their authentic self; “passing” as cisgender; finding acceptance within the LGB community; desire but struggles to have intimate relationships; conflict between internal and external sense of self; and histories of depression, and suicidality. Protective factors included having an internal source of strength; support within the T/GNC community; being in committed relationships; and receiving support from others. Age at time of transitioning, sex assigned at birth, and identifying as GNB but not transgender influenced lived experiences. McDowell, M.J., Hughto, J.M.W., & Resiner, S.L. (2019). Risk and protective factors for mental health morbidity in a community sample of female-to- male trans-masculine adults. BMC Psychiatry, 19(16), 1-12. https://doi.org/10.1186/s12888-018-2008-0 Sloan, S., & Benson, J. J. (2022). Toward a conceptual model for successful transgender aging. Qualitative Social Work, 21(2), 455-471. https://doi.org/10.1177/1473325021994666

